# Concentration and Quantification of SARS-CoV-2 RNA in Wastewater Using Polyethylene Glycol-Based Concentration and qRT-PCR

**DOI:** 10.3390/mps4010017

**Published:** 2021-02-23

**Authors:** Kata Farkas, Luke S. Hillary, Jamie Thorpe, David I. Walker, James A. Lowther, James E. McDonald, Shelagh K. Malham, Davey L. Jones

**Affiliations:** 1 School of Natural Sciences, Bangor University, Deiniol Road, Bangor LL57 2UW, UK; luke.hillary@bangor.ac.uk (L.S.H.); osp82d@bangor.ac.uk (J.T.); j.mcdonald@bangor.ac.uk (J.E.M.); d.jones@bangor.ac.uk (D.L.J.); 2 School of Ocean Sciences, Bangor University, Menai Bridge LL59 5AB, UK; s.malham@bangor.ac.uk; 3 UK National Reference Laboratory for Foodborne Viruses, Centre for Environment, Fisheries and Aquaculture Science, Weymouth DT4 8UB, UK; david.walker@cefas.co.uk (D.I.W.); james.lowther@cefas.co.uk (J.A.L.); 4 UWA School of Agriculture and Environment, The University of Western Australia, Perth, WA 6009, Australia

**Keywords:** COVID-19, environmental samples, sewage, wastewater virology, public health

## Abstract

Wastewater-based epidemiology has become an important tool for the surveillance of SARS-CoV-2 outbreaks. However, the detection of viruses in sewage is challenging and to date there is no standard method available which has been validated for the sensitive detection of SARS-CoV-2. In this paper, we describe a simple concentration method based on polyethylene glycol (PEG) precipitation, followed by RNA extraction and a one-step quantitative reverse transcription PCR (qRT-PCR) for viral detection in wastewater. PEG-based concentration of viruses is a simple procedure which is not limited by the availability of expensive equipment and has reduced risk of disruption to consumable supply chains. The concentration and RNA extraction steps enable 900–1500× concentration of wastewater samples and sufficiently eliminates the majority of organic matter, which could inhibit the subsequent qRT-PCR assay. Due to the high variation in the physico-chemical properties of wastewater samples, we recommend the use of process control viruses to determine the efficiency of each step. This procedure enables the concentration and the extraction the DNA/RNA of different viruses and hence can be used for the surveillance of different viral targets for the comprehensive assessment of viral diseases in a community.

## 1. Introduction

Wastewater-based epidemiology (WBE) is an important tool for the surveillance of viral diseases at a community level [1]. Studies on the temporal monitoring of enteric viruses in sewage have shown good correlation between viral titers in samples and local registered illnesses and outbreaks [2,3,4]. Since March 2020, WBE has also been used for the surveillance of COVID-19 [5]. Research has shown that the SARS-CoV-2 concentrations in sewage correlates with the number of infection cases and that the virus can be detected in wastewater even before the first cases are diagnosed [6]. 

However, while WBE represents a powerful tool to monitor the temporal and spatial dynamics of viral diseases, the detection of viruses in complex matrices, such as treated and untreated wastewater remains challenging. Prior to analysis, RNA viruses in wastewater are typically concentrated, followed by the extraction and reverse transcription (RT) of RNA and PCR-based quantification, which enables the accurate and sensitive detection of viruses at genus/species/genogroup/genotype/strain level. To date, methods, including ultrafiltration, ultracentrifugation, filtration-adsorption, flocculation and polyethylene glycol (PEG) precipitation, commonly used for the detection of enteric viruses have been implemented for the detection of SARS-CoV-2 [7,8]. However, in most cases the efficiency of these methods for recovering and quantifying enveloped virus concentrations have not been assessed.

This protocol describes an efficient method for the concentration of viruses from wastewater and the extraction and quantification of viral RNA, enabling the effective monitoring of SARS-CoV-2. The recommended start volume is 100–200 mL of untreated wastewater, and the final volume is 0.1 mL. Wastewater concentration starts with a centrifugation step for the elimination of large particulate matter. After pH adjustment, where necessary, the supernatant is incubated in a PEG8000/NaCl solution and the viruses bound to PEG are pelleted via centrifugation. The resulting pellet is resuspended, and the viral RNA is extracted and quantified using a one-step quantitative RT-PCR (qRT-PCR) assay. The triplex qRT-PCR described here is suitable for the detection of three targets, including the SARS-CoV-2 N1 and E genes and an extraction control, murine norovirus (MNV) or a whole process control, the porcine respiratory and reproductive syndrome virus (PRRSV). 

## 2. Experimental Design

### 2.1. Materials

- PEG8000 (Sigma Aldrich, St. Louis, MO, USA, Cat. No. P5413).
- NaCl (Sigma Aldrich, St. Louis, MO, USA, Cat. No. S7653).
- Deionised water or reverse osmosis water with purity ≥18 MΩ resistance.
- Sterile phosphate buffered saline (PBS), pH 7.4 (Sigma Aldrich, St. Louis, MO, USA, Cat. No. P4417).
- 1 M NaOH and 0.5 M HCl for pH adjustment.
- pH 6.0–10.0 pH strips (VWR, Radnor, PA, USA, Cat No. 85414.601P) or equivalent if pH meter is not available.
- NucliSens lysis buffer (BioMerieux, Marcy-l’Étoile, France, Cat No. 280,134 or 200292).
- NucliSens extraction reagent kit (BioMerieux, Marcy-l’Étoile, France, Cat. No. 200293), containing:
  ○ Magnetic beads mix (Cat No. 280133)
  ○ Wash Buffer 1 (Cat No. 280130)
  ○ Wash Buffer 2 (Cat No. 280131)
  ○ Wash Buffer 3 (Cat No. 280132)
  ○ Elution Buffer (Cat No. 280132)
    ■ Elution buffer and Wash Buffer 3 are the same reagents which are aliquoted in separate tubes in the extraction reagent kit.
- Optional control viruses:
  ○ Whole process control: non-human enveloped virus stock, e.g., PRRSV (American Type Culture Collection (ATCC), Manassas, VA, USA, Cat. No. VR-2385).
  ○ RNA extraction control: non-human RNA virus stock, e.g., MNV (ATCC, Cat. No. VR-1937).
- RNA UltraSense™ One-Step Quantitative RT-PCR System (Life Technologies, Carlsbad, CA, USA, Cat. No. 11732927), containing:
  ○ 5× reaction mix
  ○ 20× enzyme mix
  ○ 20× bovine serum albumin (BSA)
  ○ 50 nM MgSO_4_
  ○ ROX reference dye
- qPCR primers and probes as detailed in Table 1.
- DNA standards incorporating the target sequences or equivalent:
  ○ SARS-CoV-2 N1: 2019-nCoV_N_positive control (Integrated DNA Technologies (IDT), Coralville, IA, USA, Cat. No. 10006625)
  ○ SARS-CoV-2 E: 2019-nCoV_E control (IDT, Coralville, IA, USA, Cat. No. N/A)
  ○ MNV/PRRSV: custom Ultramer DNA oligo (IDT, Coralville, IA, USA)

### 2.2. Equipment

- Temperature-controlled benchtop centrifuge with fixed-angle rotor and sealed buckets with 50–200 mL tube inserts and capable of speeds up to 10,000× *g* (Eppendorf, Hamburg, Germany).
- Temperature-controlled benchtop centrifuge with swinging-bucket rotor and sealed buckets with 50–200 mL tube inserts and capable of speeds up to 3000× *g* (Eppendorf, Hamburg, Germany).
- pH meter, pocket size (Ichiro Corporation, Kotoku, Tokyo, Japan, Cat. No. S2K992) or equivalent.
- Microbiological Safety Cabinet (CL2/BSL2 compliant).
- Vortex mixer.
- Thermoshaker operating at 60 °C and 1400 rpm or equivalent.
- Magnetic rack for 1.5 mL tubes.
- Optional: NucliSens MiniMAG instrument or other (semi-)automated liquid handling system (BioMerieux, Marcy-l’Étoile, France).
- Optional: aspirator or equivalent apparatus for removing supernatant.
- Quant Studio Flex 6 real-time PCR machine (Applied Biosystems Inc., Waltham, MA, USA) or equivalent (real-time PCR machine with minimum four channels).

## 3. Procedure

**Figure 1:**
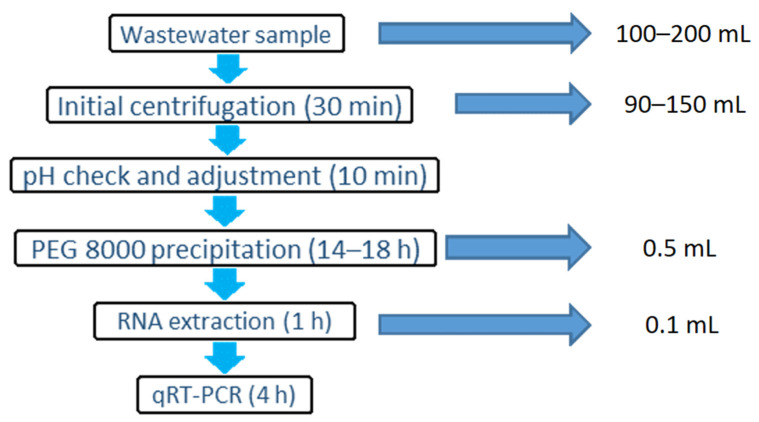
The stages of the wastewater concentration, RNA extraction and quantification method for SARS-CoV-2.

The following steps (summarized in Figure 1) can be performed in a Containment Level 2 (CL2)/Biosafety Level 2 (BSL2) environment following WHO [13] and national biosafety guidelines. The use of face masks and/or visors is advised during sample concentration, extraction and quantification preparations to minimize the chance of contamination. 

### 3.1. Samples and Controls

- Samples: 100/200 mL untreated wastewater. Samples should be delivered to laboratory chilled within 24 h of sampling. Polypropylene bottles are recommended for sample collection and shipping. Sample volume can be reduced or increase based on the turbidity of the sample.
- Process negative control: 100/200 mL sterile water.
- Process positive control: 90/150 mL sterile water spiked with approx. 10^5^ gc PRRSV or equivalent.
- Extraction negative control: 0.5 mL PBS.
- Extraction positive control: 0.5 mL PBS spiked with approx. 10^5^ gc MNV or equivalent.
- qRT-PCR non-template control (NTC): 10 µL molecular-grade water.

### 3.2. Sample Concentration. Time for Completion: 20–28 h, Bench Time: 1.5 h

We use a Microbiological Safety Cabinet (CL2/BSL2) to perform steps where the sample containers and rotors are open.

1. Aliquot 100/200 mL per wastewater sample into individual 50 mL polypropylene centrifuge tubes. Use one negative control in each batch of samples (100–200 mL water) and treat as a sample.
2. Centrifuge samples at 3000× g at 4 °C for 30 min. Alternatively, a 10-min centrifugation at 10,000× g at 4 °C can be used. Adjust break speed to 0–5 to avoid resuspension of the pellet.
3. Remove and combine 90/150 mL of the supernatant in a new sterile 250 mL polypropylene bottle. Do not disturb the pellet. The volume of liquid may be changed, depending on the amount of pellet. Discard pellet.
4. Optional step: spike supernatant with approx. 10^5^ gc equivalent PRRSV. Spike 90/150 mL sterile water as well as a positive control.
5. Adjust the pH of the 90/150 mL supernatant to 7–7.5 (usually up to 1–3 drops of 1 M NaOH) to enhance protein binding.
6. Add 1:3 ratio of 40% PEG 8000, 8% NaCl solution (as described in Section 5) to each sample (pH checked) to reach a final concentration of 10% PEG 8000, 2% NaCl. Invert several times to mix.
7. Incubate samples at 4 °C for 14–18 h.
8. Make a mark on the tube on the side where the pellet will form as it is often difficult to see. Alternatively, place the tubes in the centrifuge with label facing up. Keep the tube in same orientation across all centrifuge steps to ensure the pellet builds up in the same location.
9. Centrifuge at 10,000× g for 30 min at 4 °C. Adjust break speed to 0–5 to avoid the resuspension of the pellet.
10. Discard supernatant by decanting and pipetting.
11. Thoroughly resuspend the pellet in 0.5 mL PBS and proceed with nucleic acid extraction or store the concentrate at 4 °C for up to three days, −20° for seven days or −80 °C for long term. Alternatively, 2 mL Nuclisens lysis buffer may be added directly to the pellet then either extract immediately or store at −80 °C. However, mixing the pellet with the buffer may result in excessive foaming.

### 3.3. Viral RNA Extraction. Time for Completion: 1.5 h, Bench Time: 1.5 h

Use a Microbiological Safety Cabinet to perform Steps 1–5.

1. Optional step: add approx. 10^5^ gc equivalent murine norovirus control to each sample. Each day of extractions, include one negative (MNV- 0.5 mL water) and one positive control (MNV+ 0.5 mL water spiked with MNV).
2. Add 2 mL of NucliSens lysis buffer to a 15 mL centrifuge tube. Add 500 μL of sample and mix by vortexing (skip this step if lysis buffer was added directly to the pellet in step 3.2.10).
3. Incubate for 10 min at room temperature.
4. Add 50 μL of well-mixed magnetic bead solution from the extraction kit to the tube and mix by vortexing briefly.
5. Incubate for 10 min at room temperature.
6. Centrifuge for 2 min at 1500× g then carefully discard supernatant by pipetting or aspiration.
7. Add 385 μL wash buffer 1 and resuspend the pellet by pipetting/vortexing.
8. Transfer suspension to a 1.5 mL centrifuge tube, vortex for 30 s at low speed and separate the beads using a magnetic rack.
9. Alternatively, when the MiniMag system is used, cut the tube caps off or usescrew-cap tubes and perform a wash using preset ‘C1 continuous’ or ‘0.5 step’ for 30 s using the MiniMAG extraction systems or by vortexing.
10. After washing, allow silica beads to settle using a magnetic rack or by raising the magnet of the MiniMAG extraction system. Discard supernatant by pipetting or aspiration.
11. Separate tubes from the magnet, then add 385 μL Wash Buffer 1. Resuspend the pellet, wash for 30 s (Step 8 or 9), allow silica beads to settle using the magnet then discard supernatant.
12. Separate tubes from the magnet, then add 485 μL Wash Buffer 2. Resuspend pellet, wash for 30 s (Step 8 or 9), allow silica beads to settle using magnet then discard supernatant. Repeat.
13. Separate tubes from magnet, then add 500 μL Wash Buffer 3. Wash for 15 s (step 8), allow silica beads to settle using magnet then discard supernatant.
  Note: samples should not be left in Wash Buffer 3 for longer than strictly necessary.
14. Add 100 μL elution buffer. Cap tubes and transfer to thermoshaker or equivalent.
15. Incubate for 5 min at 60 °C with shaking at 1400 rpm.
16. Place tubes in magnetic rack and allow silica beads to settle, then transfer eluate to a clean, labelled 0.5 mL tube. Continue to qRT-PCR or store the extracts at −80 °C.

### 3.4. qRT-PCR Quantification of Viral Genome Copies. Time for Completion: 4.5 h, Bench Time: 1–1.5 h

1. Prepare and aliquot primer and probe mixes to avoid repeated freezing/thawing.
2. Prepare qRT-PCR master mix as described in Table 2. Each sample should be run in duplicate. Duplicates of standard dilution series (at least five points in the concentration range of 10^6^–10^0^ gc/µL) and of a no template control (NTC) consisting of molecular biology grade water or Tris-EDTA (TE) buffer should be included in each run.
3. Use run conditions described in Table 3 for the one-step qRT-PCR reaction.

### 3.5. Data Analysis

SARS-CoV-2 concentrations should be calculated as:

N1 or E gene/mL wastewater = gc number in qRT-PCR reaction × 20/sample supernatant volume in mL(1)

MNV/PRRSV recoveries should be calculated as:

Recovery % = gc in sample/gc in positive control × 100(2)

Quality control:

- Process negative control, extraction negative control and NTC should be negative for all gene targets.
- qRT-PCR standard curve should be in line with the recommendations described in The MIQE Guidelines [14].
- Ct values > 40 should be considered negative. We rcommend using 45 cycles of amplification to assess the amplification curve for samples with Ct values of 38–40.
- Control virus recovery should exceed 1%, as suggested by previous standard method for viral detection in shellfish (ISO 15216-2:2019). When viral recovery is lower than 1%, the qRT-PCR should be repeated with 2 µL samples added to the reaction mix to assess potential inhibition.

## 4. Expected Results

### 4.1. Method Efficiency

We have tested the limit of detection (LOD) and limit of quantification (LOQ) of the triplex assay targeting the SARS-CoV-2 N1 and E genes and the MNV by spiking wastewater extracts with viral RNA with nominal concentrations of approx. 100, 50, 20, 10, 5 and 2 gc/µL. Replicates of ten of each dilution were then tested and quantified using a dilution series of DNA standards. The LOD was determined as the lowest concentration where all ten replicates were positive. LOQ was determined as the lowest concentration where the coefficient of variance was below 0.25. LOD and LOQ values are summarized in Table 4. Our preliminary results showed that the multiplexing had no significant effect on the amplification (Figure S1).

To assess process efficiency, we spiked 90 mL of three wastewater sample supernatants after the initial centrifugation step in duplicates/triplicates with 3.0 × 10^5^ gc PRRSV and concentrated the samples as described above. The samples are taken at different wastewater treatment plants in the UK and labelled as D, L and M for anonymity (Table 5). The concentrates were spiked with 1.9 × 10^5^ gc MNV and the samples were then extracted, and viral RNA was quantified. Results are summarized in Table 5. No contamination in any of the negative controls were found and no qRT-PCR inhibition was observed. Based on the recovery rates, the concentration and subsequent extract methods are reproduceable and reliable for the detection of the target sequences. However, recovery rates (Table 5) may vary based on the physico-chemical properties of the wastewater samples, and hence the viral concentration efficiency should be monitored.

### 4.2. Applications and Recommendations

Precipitation using PEG is a widely used method for the capture and concentration of RNA, DNA, and viruses including the pelleting of enteric DNA and RNA viruses [3,15], human coronaviruses [16,17], including SARS-CoV-2 [18] in wastewater and in other environmental samples. Previous studies where wastewater was spiked with murine hepatitis virus (an enveloped coronavirus surrogate) suggested that PEG precipitation results in up to 73% viral recovery [19,20]. However, while most published studies recommend PEG incubation times of 2–4 h at 4 °C, the longer, overnight incubation enhances the recovery of RNA [21], and hence decayed viral fragments can be recovered. This may be beneficial for wastewater-based epidemiology, where the infectivity state of the detected viruses is not relevant.

We have tested this method with several 100 mL and 200 mL wastewater samples, however, the starting volume can be easily changed by adjusting the volume of the PEG8000 solution. We have not detected qRT-PCR inhibition. However, inhibition should be assessed when the recovery of control viruses is unsatisfactory. 

We recommend the complete spatial separation of concentration, RNA extraction and quantification using separate biosafety cabinets and/or rooms to perform each stage. The use of face masks and shields in addition to biosafety cabinets is also recommended as staff members may be asymptomatic carriers of COVID-19.

The qRT-PCR method described here was optimized using the QuantStudio real-time system. When different equipment is used, the probe dyes may need to be altered. In that case, we recommend the validation of the assay and the determination of LOD/LOQ values as described in Section 4.1.

## 5. Reagents Setup

*40% PEG 8000, 8% NaCl solution*
PEG 8000: 400 g;
NaCl: 80 g;
Deionised water: 1000 mL;

Mix 100 g PEG 8000 with 400 mL water and mix on a magnetic stirrer until dissolved. Continue adding PEG 8000 in small portions until 400 g is added. Once the PEG 8000 dissolved, add 80 g NaCl mix until dissolved and top the solution up to 1000 mL. Autoclave solution to sterilize (30 min, 121 °C) and keep at room temperature for up to 12 months.

*Primer/probe mixes*
Forward primer, 100 µM: 100 µL;
Reverse primer, 100 µM: 200 µL;
Probe, 100 µM: 50 µL;
Molecular-grade water: 650 µL;

Custom-made primers and probes should be resuspended in molecular grade water if necessary, according to the manufacturer’s instructions to reach 100 µM concentration. Primers, probes and mixes should be stored at −20 °C and completely thawed before use. Mixes should be aliquoted to small portions to avoid repeated freezing-thawing.

## Figures and Tables

**Table 1:** Primers, probes and reaction conditions used for the qRT-PCR. MNV: murine norovirus; PRRSV: porcine respiratory and reproductive syndrome virus; ORF: open reading frame.

Target	Primer/Probe	Sequence (5′-3′)	Reference
SARS-CoV-2 N1 gene region	Forward primer	GACCCCAAAATCAGCGAAAT	[9]
	Reverse primer	TCTGGTTACTGCCAGTTGAATCTG	
	Probe ^1^	[FAM]ACCCCGCATTACGTTTGGTGGACC[MGB]	
SARS-CoV-2 E gene region	Forward primer	ACAGGTACGTTAATAGTTAATAGCGT	[10]
	Reverse primer	ATATTGCAGCAGTACGCACACA	
	Probe ^1^	[VIC]ACACTAGCCATCCTTACTGCGCTTCG[QSY]	
MNV ORF1-2	Forward primer	CCGCAGGAACGCTCAGCAG	[11]
	Reverse primer	GGYTGAATGGGGACGGCCTG	
	Probe ^1^	[ABY]ATGAGTGATGGCGCA[QSY]	
PRRSV ORF7	Forward primer	CAGGACTTCGGAGCCTCGT	[12]
	Reverse primer	AGCAACTGGCACAGTTGATTGA	
	Probe ^1^	[ABY] ACGAGCTGTTAAACGAGGA[QSY]	

^1^ Probe reporters and quenchers may vary from reference.

**Table 2:** qRT-PCR reaction mix. Primer/probe mix compositions are detailed in Section 5.

Reagent	Concentration	μL/Reaction Mix
Water, molecular grade	-	8.35
Reaction mix	5×	5
Enzyme mix	20×	1.25
Bovine serum albumin (BSA)	20×	1.25
MgSO_4_	50 mM	0.4
Primer/probe mix, N1	5–20 μM	1.25
Primer/probe mix, E	5–20 μM	1.25
Primer/probe mix, MNV or PRRSV	5–20 μM	1.25
Sample/NTC/standard	-	5
Sum		25

**Table 3:** qRT-PCR run conditions.

Temperature	Time	Number of Cycles
55 °C	60 min	1
95 °C	5 min	1
95 °C	15 s	45
60 °C	1 min	
65 °C	1 min *	

* Data collection.

**Table 4:** Limit of detection (LOD) and limit of quantification (LOQ) for the triplex qRT-PCR assay targeting N1, E gene and MNV.

Target	LOD gc/Reaction	LOD gc/µL RNA Extract	LOQ gc/Reaction	LOQ gc/µL RNA Extract
SARS-CoV-2 N1	9	1.7	59	11.8
SARS-CoV-2 E	19	3.8	126	25.1
MNV	16	3.1	164	32.1

**Table 5:** Viral recovery in wastewater supernatant spiked with reproductive syndrome virus (PRRSV) and concentrates spiked with murine norovirus (MNV).

Wastewater Sample	PRRSV Recovery %	MNV Recovery %	N1 Gene Sewage	E Gene Sewage
D1	55.94	37.31	detected	detected
D2	31.52	32.05	detected	detected
L1	3.34	11.45	detected	not detected
L2	3.86	17.15	not detected	detected
L3	3.97	9.70	detected	detected
M1	9.81	16.43	not detected	not detected
M2	5.58	16.56	not detected	not detected
M3	3.41	13.21	not detected	not detected

## Supplementary Materials

The following are available online at https://www.mdpi.com/2409-9279/4/1/17/s1, Figure S1: amplification curves observed for the (A) N1 (singleplex: yellow curves, triplex: light green curves), (B) E (singleplex: red curves, triplex: light green curves) and (C) MNV (singleplex: green curves, triplex: light green curves).
