# Correction: Farkas et al. Concentration and Quantification of SARS-CoV-2 RNA in Wastewater Using Polyethylene Glycol-Based Concentration and qRT-PCR. *Methods Protoc.* 2021, *4*, 17

**DOI:** 10.3390/mps4040082

**Published:** 2021-11-12

**Authors:** Kata Farkas, Luke S. Hillary, Jamie Thorpe, David I. Walker, James A. Lowther, James E. McDonald, Shelagh K. Malham, Davey L. Jones

**Affiliations:** 1 School of Natural Sciences, Bangor University, Deiniol Road, Bangor LL57 2UW, UK; luke.hillary@bangor.ac.uk (L.S.H.); osp82d@bangor.ac.uk (J.T.); j.mcdonald@bangor.ac.uk (J.E.M.); d.jones@bangor.ac.uk (D.L.J.); 2 School of Ocean Sciences, Bangor University, Menai Bridge LL59 5AB, UK; s.malham@bangor.ac.uk; 3 UK National Reference Laboratory for Foodborne Viruses, Centre for Environment, Fisheries and Aquaculture Science, Weymouth DT4 8UB, UK; david.walker@cefas.co.uk (D.I.W.); james.lowther@cefas.co.uk (J.A.L.); 4 UWA School of Agriculture and Environment, The University of Western Australia, Perth, WA 6009, Australia

There was an error in the original article [1]. In Section 5, the volume of water to mix 100 g PEG 8000 was wrongly written as 800 mL. A correction has been made to Section 5:

Mix 100 g PEG 8000 with 400 mL water and mix on a magnetic stirrer until dissolved.

The authors apologize for any inconvenience caused and state that the scientific conclusions are unaffected. The original article has been updated.
