# Supplementary material for: Concentration and Quantification of SARS-CoV-2 RNA in Wastewater Using Polyethylene Glycol-Based Concentration and qRT-PCR
Source: Methods Protoc. 2021 Feb 23;4(1):17. doi: 10.3390/mps4010017 (PMC8005995; doi:10.3390/mps4010017)
Supplement: Supplementary file 1 [file mps-04-00017-s001.pdf]

Supplementary Information

Concentration and quantification of SARS-CoV-2 RNA in wastewater using polyethylene glycol-based concentration and qRT-PCR

Kata Farkas 1, 2*, Luke S. Hillary 1, Jamie Thorpe 1, 2, David I. Walker3, James A. Lowther 3, James E. McDonald 1, Shelagh K. Malham 2 and Davey L. Jones 1, 4

1 School of Natural Sciences, Bangor University, Deiniol Road, Bangor, Gwynedd, UK
2 School of Ocean Sciences, Bangor University, Menai Bridge, Anglesey, UK
3 UK National Reference Laboratory for Foodborne Viruses, Centre for Environment, Fisheries and Aquaculture Science, Weymouth, UK
4 UWA School of Agriculture and Environment, The University of Western Australia, Perth, WA 6009, Australia
* Correspondence: fkata211@gmail.com

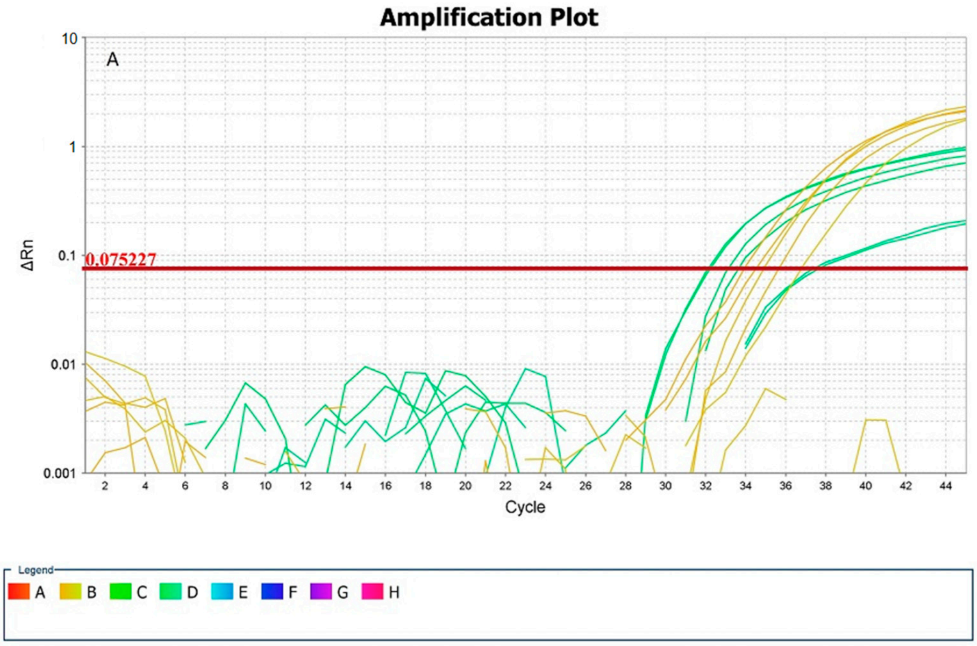

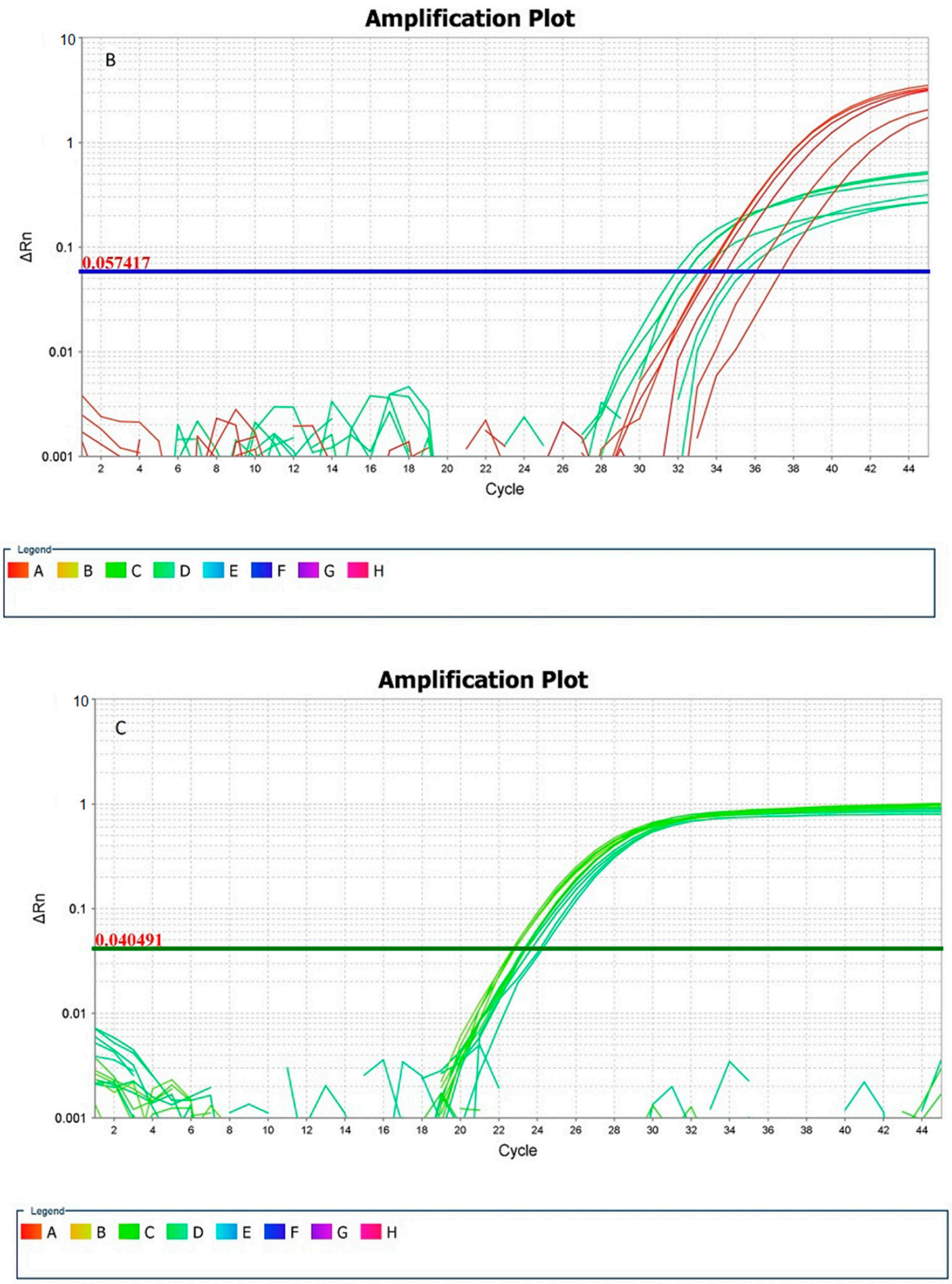

Figure S1. Amplification curves observed for the (A) N1 (singleplex: yellow curves, triplex: light green curves), (B) E (singleplex: red curves, triplex: light green curves) and (C) MNV (singleplex: green curves, triplex: light green curves).
